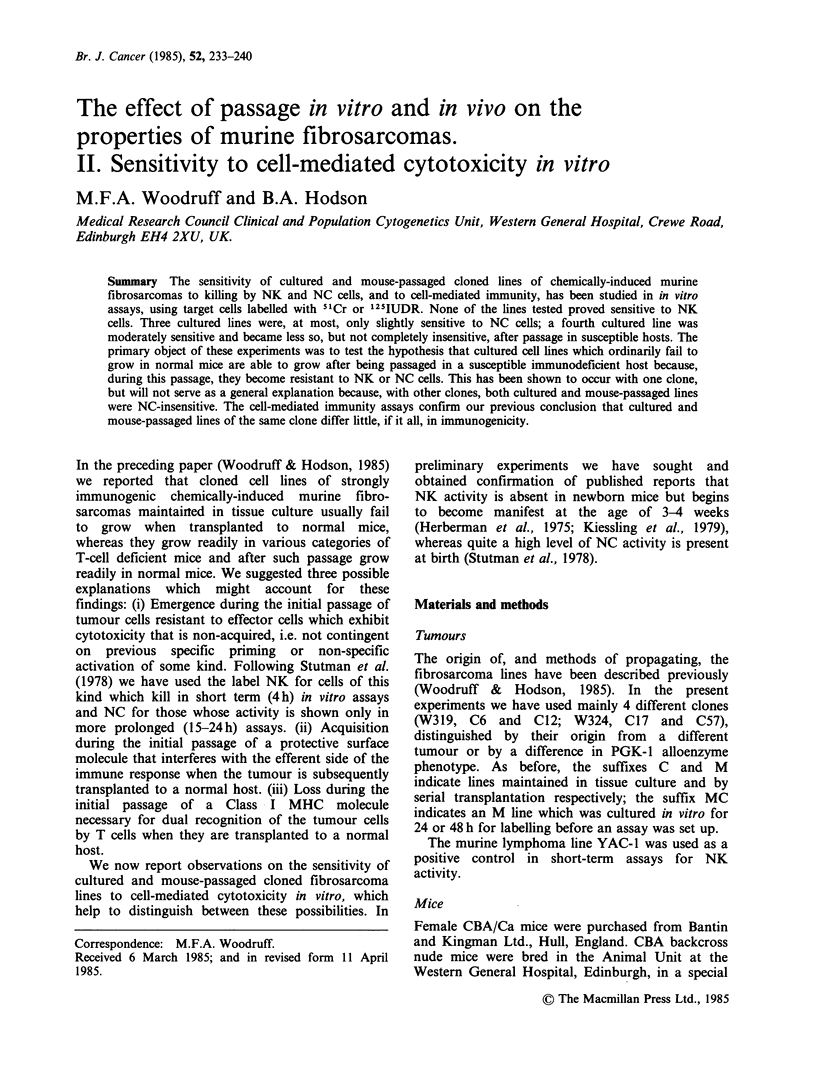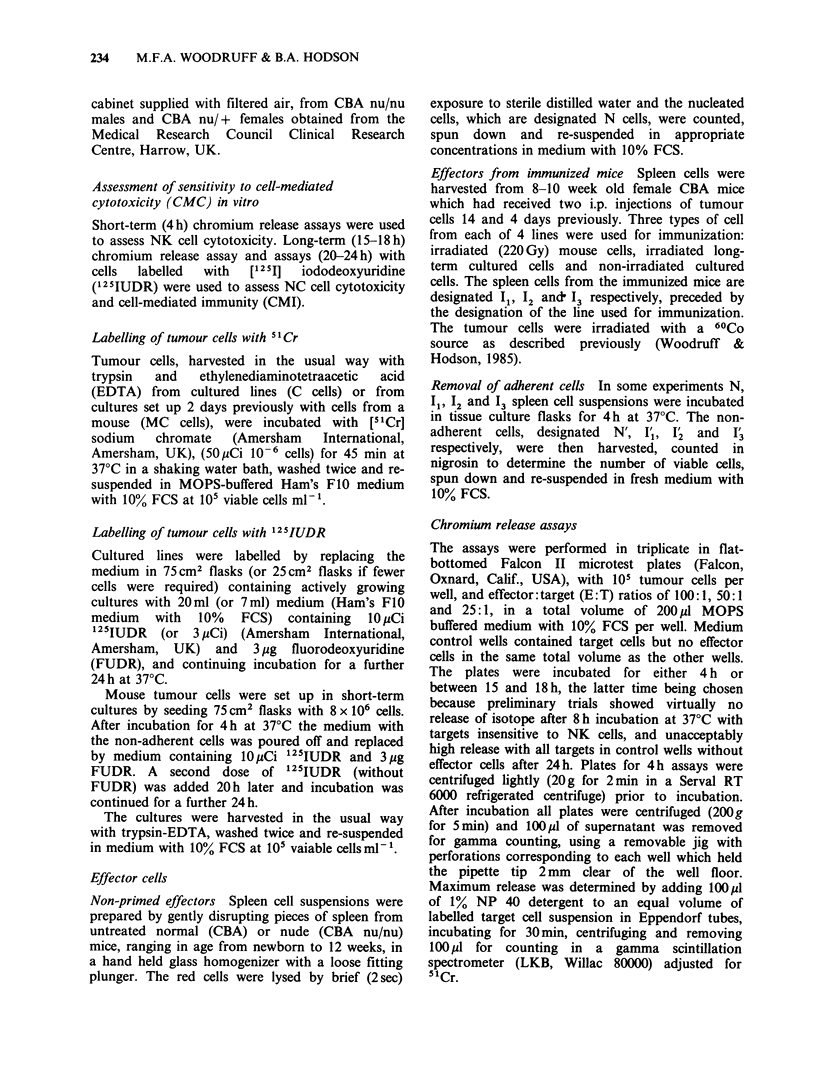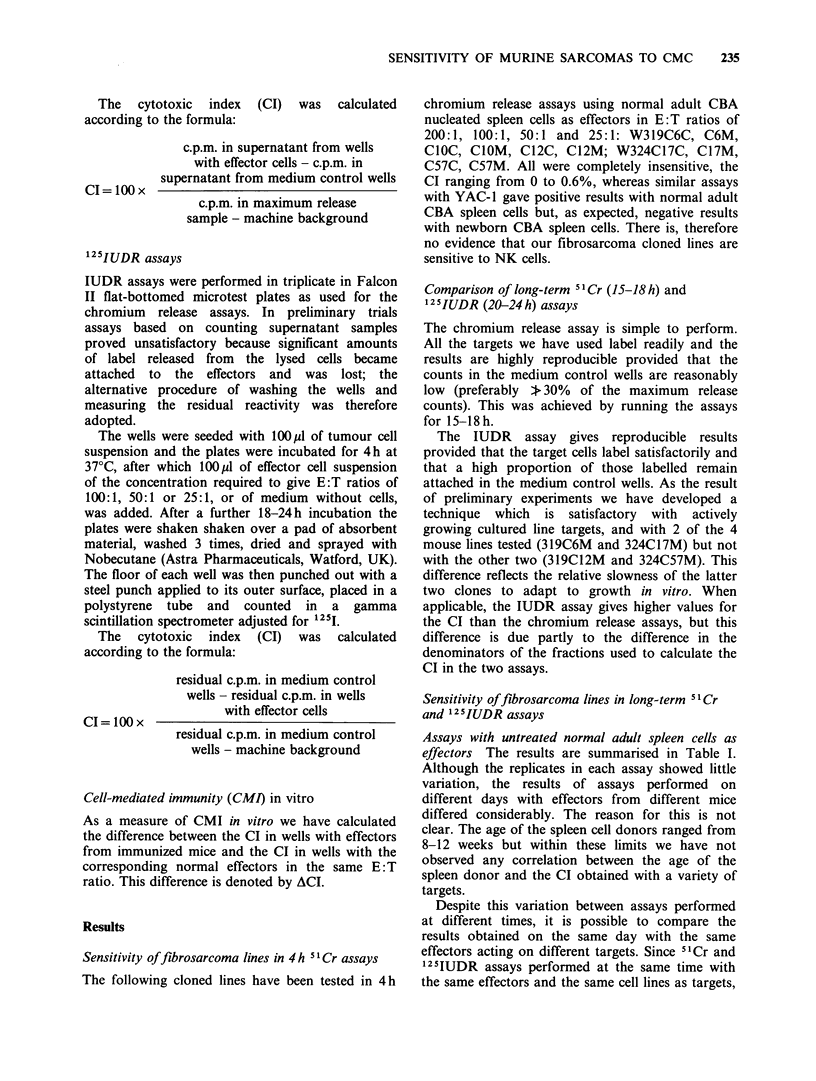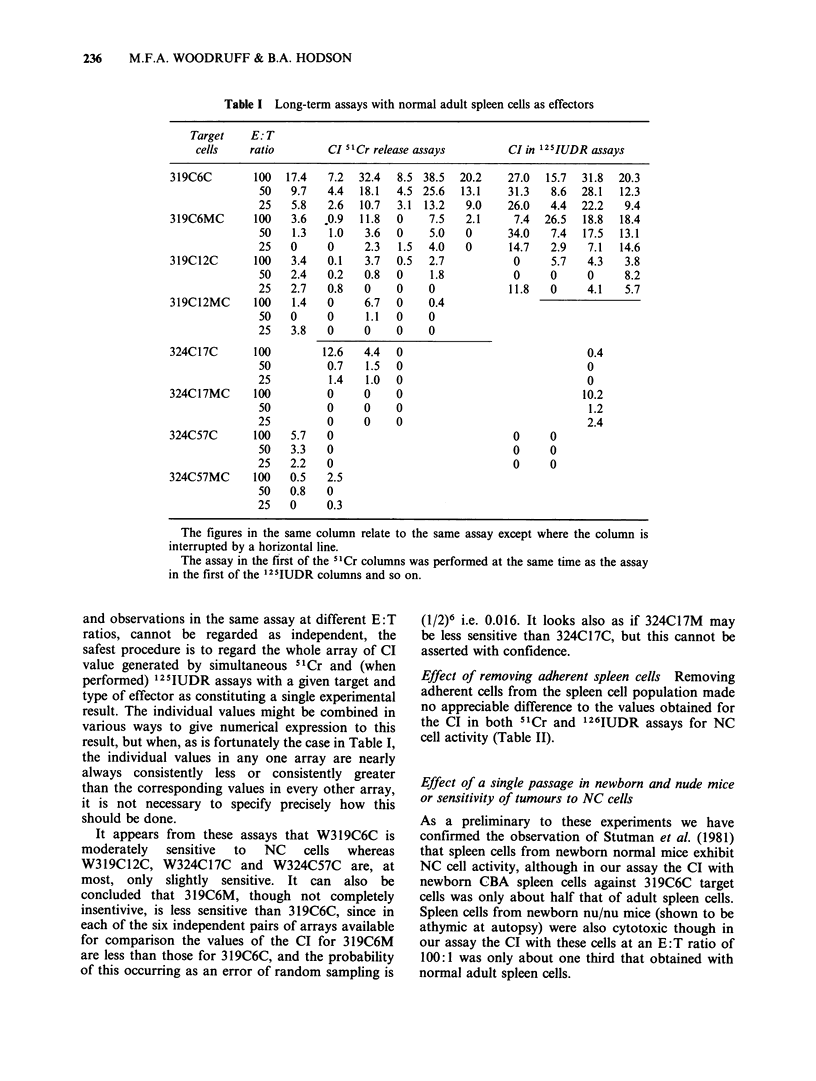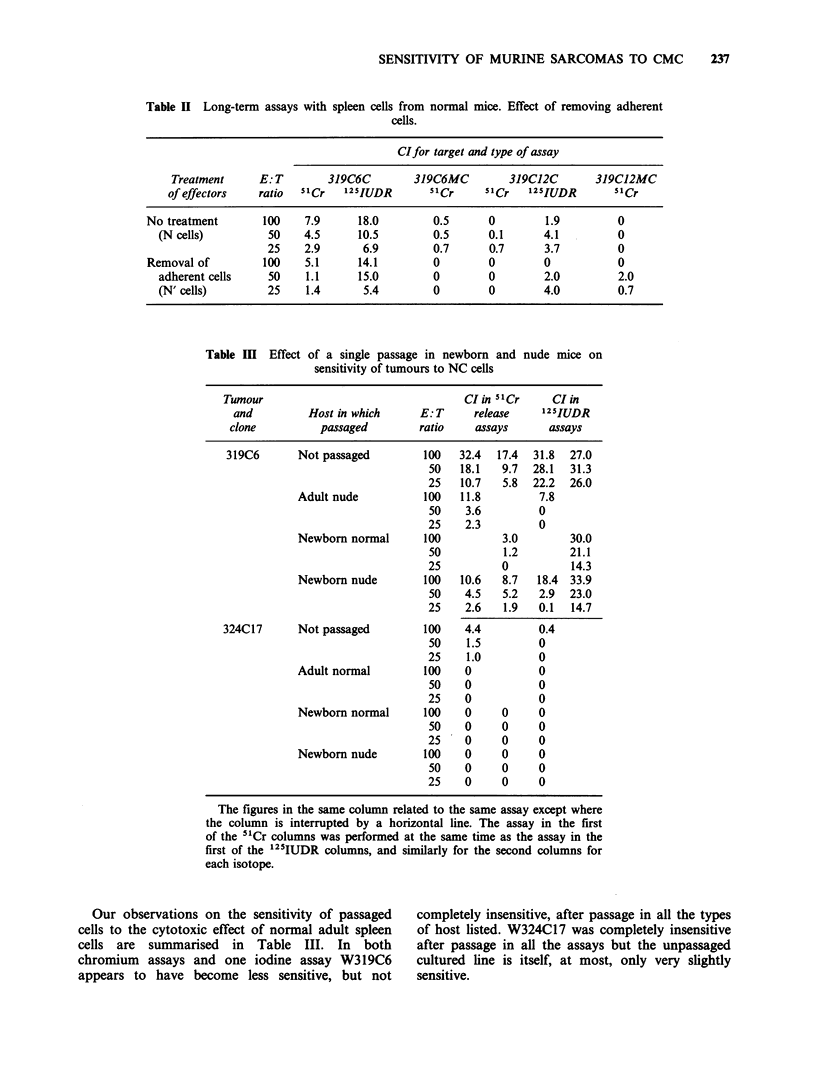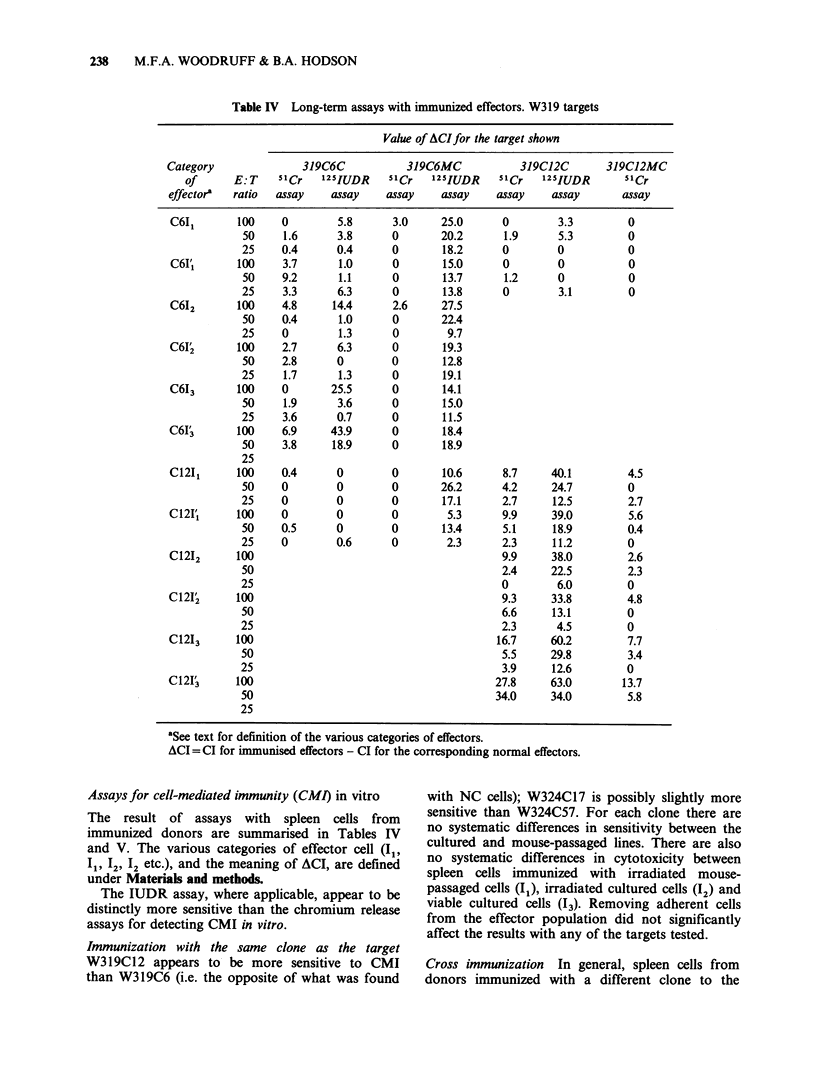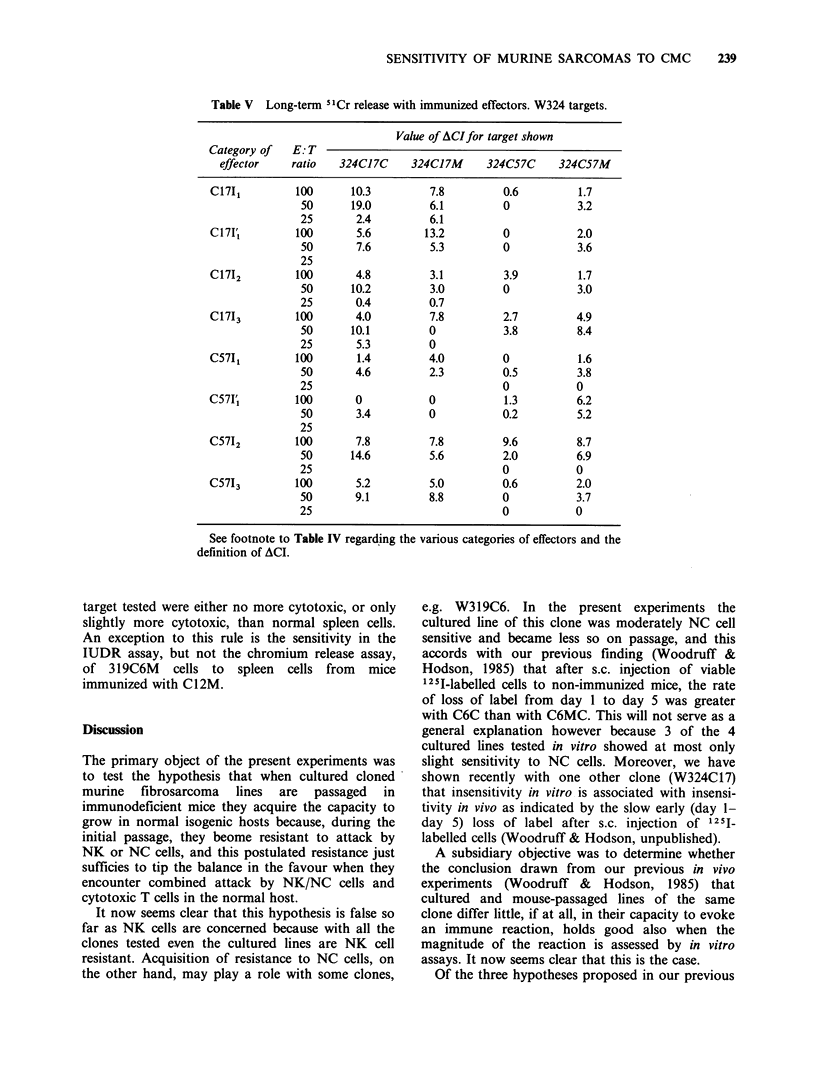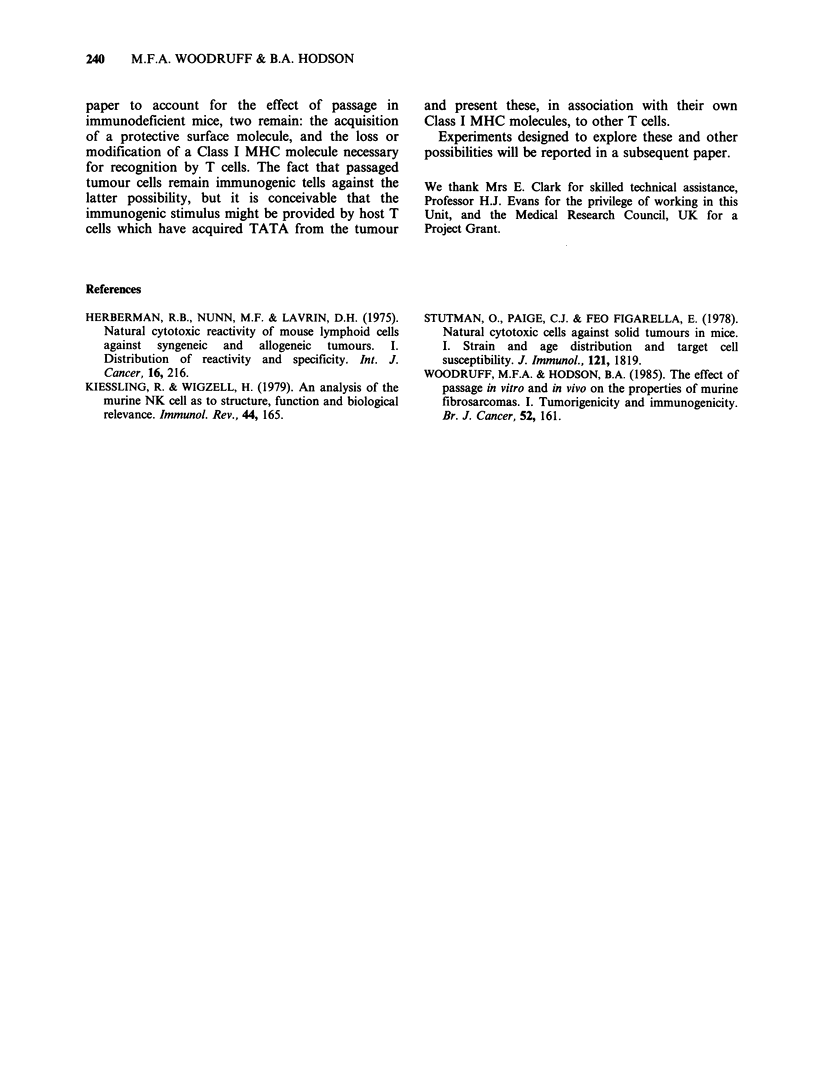# The effect of passage in vitro and in vivo on the properties of murine fibrosarcomas. II. Sensitivity to cell-mediated cytotoxicity in vitro.

**DOI:** 10.1038/bjc.1985.182

**Published:** 1985-08

**Authors:** M. F. Woodruff, B. A. Hodson

## Abstract

The sensitivity of cultured and mouse-passaged cloned lines of chemically-induced murine fibrosarcomas to killing by NK and NC cells, and to cell-mediated immunity, has been studied in in vitro assays, using target cells labelled with 51Cr or 125IUDR. None of the lines tested proved sensitive to NK cells. Three cultured lines were, at most, only slightly sensitive to NC cells; a fourth cultured line was moderately sensitive and became less so, but not completely insensitive, after passage in susceptible hosts. The primary object of these experiments was to test the hypothesis that cultured cell lines which ordinarily fail to grow in normal mice are able to grow after being passaged in a susceptible immunodeficient host because, during this passage, they become resistant to NK or NC cells. This has been shown to occur with one clone, but will not serve as a general explanation because, with other clones, both cultured and mouse-passaged lines were NC-insensitive. The cell-mediated immunity assays confirm our previous conclusion that cultured and mouse-passaged lines of the same clone differ little, if it all, in immunogenicity.


*Br. J. Cancer* (1985), **52**, 233–240

### The effect of passage *in vitro* and *in vivo* on the properties of murine fibrosarcomas. II. Sensitivity to cell-mediated cytotoxicity *in vitro*

M.F.A. Woodruff and B.A. Hodson

*Medical Research Council Clinical and Population Cytogenetics Unit, Western General Hospital, Crewe Road, Edinburgh EH4 2XU, UK.*

**Summary** The sensitivity of cultured and mouse-passaged cloned lines of chemically-induced murine fibrosarcomas to killing by NK and NC cells, and to cell-mediated immunity, has been studied in *in vitro* assays, using target cells labelled with $^{51}$Cr or $^{125}$IUDR. None of the lines tested proved sensitive to NK cells. Three cultured lines were, at most, only slightly sensitive to NC cells; a fourth cultured line was moderately sensitive and became less so, but not completely insensitive, after passage in susceptible hosts. The primary object of these experiments was to test the hypothesis that cultured cell lines which ordinarily fail to grow in normal mice are able to grow after being passaged in a susceptible immunodeficient host because, during this passage, they become resistant to NK or NC cells. This has been shown to occur with one clone, but will not serve as a general explanation because, with other clones, both cultured and mouse-passaged lines were NC-insensitive. The cell-mediated immunity assays confirm our previous conclusion that cultured and mouse-passaged lines of the same clone differ little, if it all, in immunogenicity.

In the preceding paper (Woodruff & Hodson, 1985) we reported that cloned cell lines of strongly immunogenic chemically-induced murine fibrosarcomas maintained in tissue culture usually fail to grow when transplanted to normal mice, whereas they grow readily in various categories of T-cell deficient mice and after such passage grow readily in normal mice. We suggested three possible explanations which might account for these findings: (i) Emergence during the initial passage of tumour cells resistant to effector cells which exhibit cytotoxicity that is non-acquired, i.e. not contingent on previous specific priming or non-specific activation of some kind. Following Stutman *et al.* (1978) we have used the label NK for cells of this kind which kill in short term (4 h) *in vitro* assays and NC for those whose activity is shown only in more prolonged (15–24 h) assays. (ii) Acquisition during the initial passage of a protective surface molecule that interferes with the efferent side of the immune response when the tumour is subsequently transplanted to a normal host. (iii) Loss during the initial passage of a Class I MHC molecule necessary for dual recognition of the tumour cells by T cells when they are transplanted to a normal host.

We now report observations on the sensitivity of cultured and mouse-passaged cloned fibrosarcoma lines to cell-mediated cytotoxicity *in vitro,* which help to distinguish between these possibilities. In preliminary experiments we have sought and obtained confirmation of published reports that NK activity is absent in newborn mice but begins to become manifest at the age of 3–4 weeks (Herberman *et al.,* 1975; Kiessling *et al.,* 1979), whereas quite a high level of NC activity is present at birth (Stutman *et al.,* 1978).

Correspondence: M.F.A. Woodruff.
Received 6 March 1985; and in revised form 11 April 1985.

### Materials and methods

### *Tumours*

The origin of, and methods of propagating, the fibrosarcoma lines have been described previously (Woodruff & Hodson, 1985). In the present experiments we have used mainly 4 different clones (W319, C6 and C12; W324, C17 and C57), distinguished by their origin from a different tumour or by a difference in PGK-1 alloenzyme phenotype. As before, the suffixes C and M indicate lines maintained in tissue culture and by serial transplantation respectively; the suffix MC indicates an M line which was cultured *in vitro* for 24 or 48 h for labelling before an assay was set up.

The murine lymphoma line YAC-1 was used as a positive control in short-term assays for NK activity.

### *Mice*

Female CBA/Ca mice were purchased from Bantin and Kingman Ltd., Hull, England. CBA backcross nude mice were bred in the Animal Unit at the Western General Hospital, Edinburgh, in a special

© The Macmillan Press Ltd., 1985

cabinet supplied with filtered air, from CBA nu/nu males and CBA nu/+ females obtained from the Medical Research Council Clinical Research Centre, Harrow, UK.

*Assessment of sensitivity to cell-mediated cytotoxicity (CMC) in vitro*

Short-term (4 h) chromium release assays were used to assess NK cell cytotoxicity. Long-term (15–18 h) chromium release assay and assays (20–24 h) with cells labelled with [$^{125}$I] iododeoxyuridine ($^{125}$IUDR) were used to assess NC cell cytotoxicity and cell-mediated immunity (CMI).

*Labelling of tumour cells with $^{51}$Cr*

Tumour cells, harvested in the usual way with trypsin and ethylenediaminotetraacetic acid (EDTA) from cultured lines (C cells) or from cultures set up 2 days previously with cells from a mouse (MC cells), were incubated with [$^{51}$Cr] sodium chromate (Amersham International, Amersham, UK), (50 $\mu$Ci $10^{-6}$ cells) for 45 min at 37°C in a shaking water bath, washed twice and re-suspended in MOPS-buffered Ham's F10 medium with 10% FCS at $10^5$ viable cells ml$^{-1}$.

*Labelling of tumour cells with $^{125}$IUDR*

Cultured lines were labelled by replacing the medium in 75 cm$^2$ flasks (or 25 cm$^2$ flasks if fewer cells were required) containing actively growing cultures with 20 ml (or 7 ml) medium (Ham's F10 medium with 10% FCS) containing 10 $\mu$Ci $^{125}$IUDR (or 3 $\mu$Ci) (Amersham International, Amersham, UK) and 3 $\mu$g fluorodeoxyuridine (FUDR), and continuing incubation for a further 24 h at 37°C.

Mouse tumour cells were set up in short-term cultures by seeding 75 cm$^2$ flasks with $8 \times 10^6$ cells. After incubation for 4 h at 37°C the medium with the non-adherent cells was poured off and replaced by medium containing 10 $\mu$Ci $^{125}$IUDR and 3 $\mu$g FUDR. A second dose of $^{125}$IUDR (without FUDR) was added 20 h later and incubation was continued for a further 24 h.

The cultures were harvested in the usual way with trypsin-EDTA, washed twice and re-suspended in medium with 10% FCS at $10^5$ vaiable cells ml$^{-1}$.

*Effector cells*

*Non-primed effectors* Spleen cell suspensions were prepared by gently disrupting pieces of spleen from untreated normal (CBA) or nude (CBA nu/nu) mice, ranging in age from newborn to 12 weeks, in a hand held glass homogenizer with a loose fitting plunger. The red cells were lysed by brief (2 sec) exposure to sterile distilled water and the nucleated cells, which are designated N cells, were counted, spun down and re-suspended in appropriate concentrations in medium with 10% FCS.

*Effectors from immunized mice* Spleen cells were harvested from 8–10 week old female CBA mice which had received two i.p. injections of tumour cells 14 and 4 days previously. Three types of cell from each of 4 lines were used for immunization: irradiated (220 Gy) mouse cells, irradiated long-term cultured cells and non-irradiated cultured cells. The spleen cells from the immunized mice are designated $I_1$, $I_2$ and $I_3$ respectively, preceded by the designation of the line used for immunization. The tumour cells were irradiated with a $^{60}$Co source as described previously (Woodruff & Hodson, 1985).

*Removal of adherent cells* In some experiments N, $I_1$, $I_2$ and $I_3$ spleen cell suspensions were incubated in tissue culture flasks for 4 h at 37°C. The non-adherent cells, designated N′, $I'_1$, $I'_2$ and $I'_3$ respectively, were then harvested, counted in nigrosin to determine the number of viable cells, spun down and re-suspended in fresh medium with 10% FCS.

*Chromium release assays*

The assays were performed in triplicate in flat-bottomed Falcon II microtest plates (Falcon, Oxnard, Calif., USA), with $10^5$ tumour cells per well, and effector:target (E:T) ratios of 100:1, 50:1 and 25:1, in a total volume of 200 $\mu$l MOPS buffered medium with 10% FCS per well. Medium control wells contained target cells but no effector cells in the same total volume as the other wells. The plates were incubated for either 4 h or between 15 and 18 h, the latter time being chosen because preliminary trials showed virtually no release of isotope after 8 h incubation at 37°C with targets insensitive to NK cells, and unacceptably high release with all targets in control wells without effector cells after 24 h. Plates for 4 h assays were centrifuged lightly (20 *g* for 2 min in a Serval RT 6000 refrigerated centrifuge) prior to incubation. After incubation all plates were centrifuged (200 *g* for 5 min) and 100 $\mu$l of supernatant was removed for gamma counting, using a removable jig with perforations corresponding to each well which held the pipette tip 2 mm clear of the well floor. Maximum release was determined by adding 100 $\mu$l of 1% NP 40 detergent to an equal volume of labelled target cell suspension in Eppendorf tubes, incubating for 30 min, centrifuging and removing 100 $\mu$l for counting in a gamma scintillation spectrometer (LKB, Willac 80000) adjusted for $^{51}$Cr.

The cytotoxic index (CI) was calculated according to the formula:

$$CI = 100 \times \frac{\text{c.p.m. in supernatant from wells with effector cells} - \text{c.p.m. in supernatant from medium control wells}}{\text{c.p.m. in maximum release sample} - \text{machine background}}$$

*$^{125}$IUDR assays*

IUDR assays were performed in triplicate in Falcon II flat-bottomed microtest plates as used for the chromium release assays. In preliminary trials assays based on counting supernatant samples proved unsatisfactory because significant amounts of label released from the lysed cells became attached to the effectors and was lost; the alternative procedure of washing the wells and measuring the residual reactivity was therefore adopted.

The wells were seeded with 100 $\mu$l of tumour cell suspension and the plates were incubated for 4 h at 37°C, after which 100 $\mu$l of effector cell suspension of the concentration required to give E:T ratios of 100:1, 50:1 or 25:1, or of medium without cells, was added. After a further 18–24 h incubation the plates were shaken shaken over a pad of absorbent material, washed 3 times, dried and sprayed with Nobecutane (Astra Pharmaceuticals, Watford, UK). The floor of each well was then punched out with a steel punch applied to its outer surface, placed in a polystyrene tube and counted in a gamma scintillation spectrometer adjusted for $^{125}$I.

The cytotoxic index (CI) was calculated according to the formula:

$$CI = 100 \times \frac{\text{residual c.p.m. in medium control wells} - \text{residual c.p.m. in wells with effector cells}}{\text{residual c.p.m. in medium control wells} - \text{machine background}}$$

*Cell-mediated immunity (CMI)* in vitro

As a measure of CMI *in vitro* we have calculated the difference between the CI in wells with effectors from immunized mice and the CI in wells with the corresponding normal effectors in the same E:T ratio. This difference is denoted by $\Delta$CI.

### Results

*Sensitivity of fibrosarcoma lines in 4 h $^{51}$Cr assays*

The following cloned lines have been tested in 4 h chromium release assays using normal adult CBA nucleated spleen cells as effectors in E:T ratios of 200:1, 100:1, 50:1 and 25:1: W319C6C, C6M, C10C, C10M, C12C, C12M; W324C17C, C17M, C57C, C57M. All were completely insensitive, the CI ranging from 0 to 0.6%, whereas similar assays with YAC-1 gave positive results with normal adult CBA spleen cells but, as expected, negative results with newborn CBA spleen cells. There is, therefore no evidence that our fibrosarcoma cloned lines are sensitive to NK cells.

*Comparison of long-term $^{51}$Cr (15–18 h) and $^{125}$IUDR (20–24 h) assays*

The chromium release assay is simple to perform. All the targets we have used label readily and the results are highly reproducible provided that the counts in the medium control wells are reasonably low (preferably $\not>$30% of the maximum release counts). This was achieved by running the assays for 15–18 h.

The IUDR assay gives reproducible results provided that the target cells label satisfactorily and that a high proportion of those labelled remain attached in the medium control wells. As the result of preliminary experiments we have developed a technique which is satisfactory with actively growing cultured line targets, and with 2 of the 4 mouse lines tested (319C6M and 324C17M) but not with the other two (319C12M and 324C57M). This difference reflects the relative slowness of the latter two clones to adapt to growth *in vitro*. When applicable, the IUDR assay gives higher values for the CI than the chromium release assays, but this difference is due partly to the difference in the denominators of the fractions used to calculate the CI in the two assays.

*Sensitivity of fibrosarcoma lines in long-term $^{51}$Cr and $^{125}$IUDR assays*

*Assays with untreated normal adult spleen cells as effectors* The results are summarised in Table I. Although the replicates in each assay showed little variation, the results of assays performed on different days with effectors from different mice differed considerably. The reason for this is not clear. The age of the spleen cell donors ranged from 8–12 weeks but within these limits we have not observed any correlation between the age of the spleen donor and the CI obtained with a variety of targets.

Despite this variation between assays performed at different times, it is possible to compare the results obtained on the same day with the same effectors acting on different targets. Since $^{51}$Cr and $^{125}$IUDR assays performed at the same time with the same effectors and the same cell lines as targets,

**Table I** Long-term assays with normal adult spleen cells as effectors

| *Target cells* | *E:T ratio* | *CI $^{51}$Cr release assays* | | | | | | *CI in $^{125}$IUDR assays* | | | |
|---|---|---|---|---|---|---|---|---|---|---|---|
| 319C6C | 100 | 17.4 | 7.2 | 32.4 | 8.5 | 38.5 | 20.2 | 27.0 | 15.7 | 31.8 | 20.3 |
| | 50 | 9.7 | 4.4 | 18.1 | 4.5 | 25.6 | 13.1 | 31.3 | 8.6 | 28.1 | 12.3 |
| | 25 | 5.8 | 2.6 | 10.7 | 3.1 | 13.2 | 9.0 | 26.0 | 4.4 | 22.2 | 9.4 |
| 319C6MC | 100 | 3.6 | 0.9 | 11.8 | 0 | 7.5 | 2.1 | 7.4 | 26.5 | 18.8 | 18.4 |
| | 50 | 1.3 | 1.0 | 3.6 | 0 | 5.0 | 0 | 34.0 | 7.4 | 17.5 | 13.1 |
| | 25 | 0 | 0 | 2.3 | 1.5 | 4.0 | 0 | 14.7 | 2.9 | 7.1 | 14.6 |
| 319C12C | 100 | 3.4 | 0.1 | 3.7 | 0.5 | 2.7 | | 0 | 5.7 | 4.3 | 3.8 |
| | 50 | 2.4 | 0.2 | 0.8 | 0 | 1.8 | | 0 | 0 | 0 | 8.2 |
| | 25 | 2.7 | 0.8 | 0 | 0 | 0 | | 11.8 | 0 | 4.1 | 5.7 |
| 319C12MC | 100 | 1.4 | 0 | 6.7 | 0 | 0.4 | | | | | |
| | 50 | 0 | 0 | 1.1 | 0 | 0 | | | | | |
| | 25 | 3.8 | 0 | 0 | 0 | 0 | | | | | |
| 324C17C | 100 | | 12.6 | 4.4 | 0 | | | | | 0.4 | |
| | 50 | | 0.7 | 1.5 | 0 | | | | | 0 | |
| | 25 | | 1.4 | 1.0 | 0 | | | | | 0 | |
| 324C17MC | 100 | | 0 | 0 | 0 | | | | | 10.2 | |
| | 50 | | 0 | 0 | 0 | | | | | 1.2 | |
| | 25 | | 0 | 0 | 0 | | | | | 2.4 | |
| 324C57C | 100 | 5.7 | 0 | | | | | 0 | 0 | | |
| | 50 | 3.3 | 0 | | | | | 0 | 0 | | |
| | 25 | 2.2 | 0 | | | | | 0 | 0 | | |
| 324C57MC | 100 | 0.5 | 2.5 | | | | | | | | |
| | 50 | 0.8 | 0 | | | | | | | | |
| | 25 | 0 | 0.3 | | | | | | | | |

The figures in the same column relate to the same assay except where the column is interrupted by a horizontal line.

The assay in the first of the $^{51}$Cr columns was performed at the same time as the assay in the first of the $^{125}$IUDR columns and so on.

and observations in the same assay at different E:T ratios, cannot be regarded as independent, the safest procedure is to regard the whole array of CI value generated by simultaneous $^{51}$Cr and (when performed) $^{125}$IUDR assays with a given target and type of effector as constituting a single experimental result. The individual values might be combined in various ways to give numerical expression to this result, but when, as is fortunately the case in Table I, the individual values in any one array are nearly always consistently less or consistently greater than the corresponding values in every other array, it is not necessary to specify precisely how this should be done.

It appears from these assays that W319C6C is moderately sensitive to NC cells whereas W319C12C, W324C17C and W324C57C are, at most, only slightly sensitive. It can also be concluded that 319C6M, though not completely insentivive, is less sensitive than 319C6C, since in each of the six independent pairs of arrays available for comparison the values of the CI for 319C6M are less than those for 319C6C, and the probability of this occurring as an error of random sampling is $(1/2)^6$ i.e. 0.016. It looks also as if 324C17M may be less sensitive than 324C17C, but this cannot be asserted with confidence.

*Effect of removing adherent spleen cells* Removing adherent cells from the spleen cell population made no appreciable difference to the values obtained for the CI in both $^{51}$Cr and $^{126}$IUDR assays for NC cell activity (Table II).

*Effect of a single passage in newborn and nude mice or sensitivity of tumours to NC cells*

As a preliminary to these experiments we have confirmed the observation of Stutman *et al.* (1981) that spleen cells from newborn normal mice exhibit NC cell activity, although in our assay the CI with newborn CBA spleen cells against 319C6C target cells was only about half that of adult spleen cells. Spleen cells from newborn nu/nu mice (shown to be athymic at autopsy) were also cytotoxic though in our assay the CI with these cells at an E:T ratio of 100:1 was only about one third that obtained with normal adult spleen cells.

**Table II** Long-term assays with spleen cells from normal mice. Effect of removing adherent cells.

| *Treatment of effectors* | *E:T ratio* | *CI for target and type of assay* | | | | | |
|---|---|---|---|---|---|---|---|
| | | *319C6C* | | *319C6MC* | *319C12C* | | *319C12MC* |
| | | *$^{51}Cr$* | *$^{125}IUDR$* | *$^{51}Cr$* | *$^{51}Cr$* | *$^{125}IUDR$* | *$^{51}Cr$* |
| No treatment | 100 | 7.9 | 18.0 | 0.5 | 0 | 1.9 | 0 |
| (N cells) | 50 | 4.5 | 10.5 | 0.5 | 0.1 | 4.1 | 0 |
| | 25 | 2.9 | 6.9 | 0.7 | 0.7 | 3.7 | 0 |
| Removal of | 100 | 5.1 | 14.1 | 0 | 0 | 0 | 0 |
| adherent cells | 50 | 1.1 | 15.0 | 0 | 0 | 2.0 | 2.0 |
| (N′ cells) | 25 | 1.4 | 5.4 | 0 | 0 | 4.0 | 0.7 |

**Table III** Effect of a single passage in newborn and nude mice on sensitivity of tumours to NC cells

| *Tumour and clone* | *Host in which passaged* | *E:T ratio* | *CI in $^{51}Cr$ release assays* | | *CI in $^{125}IUDR$ assays* | |
|---|---|---|---|---|---|---|
| 319C6 | Not passaged | 100 | 32.4 | 17.4 | 31.8 | 27.0 |
| | | 50 | 18.1 | 9.7 | 28.1 | 31.3 |
| | | 25 | 10.7 | 5.8 | 22.2 | 26.0 |
| | Adult nude | 100 | 11.8 | | 7.8 | |
| | | 50 | 3.6 | | 0 | |
| | | 25 | 2.3 | | 0 | |
| | Newborn normal | 100 | | 3.0 | | 30.0 |
| | | 50 | | 1.2 | | 21.1 |
| | | 25 | | 0 | | 14.3 |
| | Newborn nude | 100 | 10.6 | 8.7 | 18.4 | 33.9 |
| | | 50 | 4.5 | 5.2 | 2.9 | 23.0 |
| | | 25 | 2.6 | 1.9 | 0.1 | 14.7 |
| 324C17 | Not passaged | 100 | 4.4 | | 0.4 | |
| | | 50 | 1.5 | | 0 | |
| | | 25 | 1.0 | | 0 | |
| | Adult normal | 100 | 0 | | 0 | |
| | | 50 | 0 | | 0 | |
| | | 25 | 0 | | 0 | |
| | Newborn normal | 100 | 0 | 0 | 0 | |
| | | 50 | 0 | 0 | 0 | |
| | | 25 | 0 | 0 | 0 | |
| | Newborn nude | 100 | 0 | 0 | 0 | |
| | | 50 | 0 | 0 | 0 | |
| | | 25 | 0 | 0 | 0 | |

The figures in the same column related to the same assay except where the column is interrupted by a horizontal line. The assay in the first of the $^{51}$Cr columns was performed at the same time as the assay in the first of the $^{125}$IUDR columns, and similarly for the second columns for each isotope.

Our observations on the sensitivity of passaged cells to the cytotoxic effect of normal adult spleen cells are summarised in Table III. In both chromium assays and one iodine assay W319C6 appears to have become less sensitive, but not completely insensitive, after passage in all the types of host listed. W324C17 was completely insensitive after passage in all the assays but the unpassaged cultured line is itself, at most, only very slightly sensitive.

**Table IV** Long-term assays with immunized effectors. W319 targets

| | | *Value of ΔCI for the target shown* | | | | | | |
|---|---|---|---|---|---|---|---|---|
| | | *319C6C* | | *319C6MC* | | *319C12C* | | *319C12MC* |
| *Category of effector*[a] | *E:T ratio* | $^{51}Cr$ *assay* | $^{125}IUDR$ *assay* | $^{51}Cr$ *assay* | $^{125}IUDR$ *assay* | $^{51}Cr$ *assay* | $^{125}IUDR$ *assay* | $^{51}Cr$ *assay* |
| $C6I_1$ | 100 | 0 | 5.8 | 3.0 | 25.0 | 0 | 3.3 | 0 |
| | 50 | 1.6 | 3.8 | 0 | 20.2 | 1.9 | 5.3 | 0 |
| | 25 | 0.4 | 0.4 | 0 | 18.2 | 0 | 0 | 0 |
| $C6I'_1$ | 100 | 3.7 | 1.0 | 0 | 15.0 | 0 | 0 | 0 |
| | 50 | 9.2 | 1.1 | 0 | 13.7 | 1.2 | 0 | 0 |
| | 25 | 3.3 | 6.3 | 0 | 13.8 | 0 | 3.1 | 0 |
| $C6I_2$ | 100 | 4.8 | 14.4 | 2.6 | 27.5 | | | |
| | 50 | 0.4 | 1.0 | 0 | 22.4 | | | |
| | 25 | 0 | 1.3 | 0 | 9.7 | | | |
| $C6I'_2$ | 100 | 2.7 | 6.3 | 0 | 19.3 | | | |
| | 50 | 2.8 | 0 | 0 | 12.8 | | | |
| | 25 | 1.7 | 1.3 | 0 | 19.1 | | | |
| $C6I_3$ | 100 | 0 | 25.5 | 0 | 14.1 | | | |
| | 50 | 1.9 | 3.6 | 0 | 15.0 | | | |
| | 25 | 3.6 | 0.7 | 0 | 11.5 | | | |
| $C6I'_3$ | 100 | 6.9 | 43.9 | 0 | 18.4 | | | |
| | 50 | 3.8 | 18.9 | 0 | 18.9 | | | |
| | 25 | | | | | | | |
| $C12I_1$ | 100 | 0.4 | 0 | 0 | 10.6 | 8.7 | 40.1 | 4.5 |
| | 50 | 0 | 0 | 0 | 26.2 | 4.2 | 24.7 | 0 |
| | 25 | 0 | 0 | 0 | 17.1 | 2.7 | 12.5 | 2.7 |
| $C12I'_1$ | 100 | 0 | 0 | 0 | 5.3 | 9.9 | 39.0 | 5.6 |
| | 50 | 0.5 | 0 | 0 | 13.4 | 5.1 | 18.9 | 0.4 |
| | 25 | 0 | 0.6 | 0 | 2.3 | 2.3 | 11.2 | 0 |
| $C12I_2$ | 100 | | | | | 9.9 | 38.0 | 2.6 |
| | 50 | | | | | 2.4 | 22.5 | 2.3 |
| | 25 | | | | | 0 | 6.0 | 0 |
| $C12I'_2$ | 100 | | | | | 9.3 | 33.8 | 4.8 |
| | 50 | | | | | 6.6 | 13.1 | 0 |
| | 25 | | | | | 2.3 | 4.5 | 0 |
| $C12I_3$ | 100 | | | | | 16.7 | 60.2 | 7.7 |
| | 50 | | | | | 5.5 | 29.8 | 3.4 |
| | 25 | | | | | 3.9 | 12.6 | 0 |
| $C12I'_3$ | 100 | | | | | 27.8 | 63.0 | 13.7 |
| | 50 | | | | | 34.0 | 34.0 | 5.8 |
| | 25 | | | | | | | |

[a]See text for definition of the various categories of effectors.
ΔCI = CI for immunised effectors – CI for the corresponding normal effectors.

*Assays for cell-mediated immunity (CMI)* in vitro

The result of assays with spleen cells from immunized donors are summarised in Tables IV and V. The various categories of effector cell ($I_1$, $I_1$, $I_2$, $I_2$ etc.), and the meaning of ΔCI, are defined under **Materials and methods.**

The IUDR assay, where applicable, appear to be distinctly more sensitive than the chromium release assays for detecting CMI *in vitro.*

*Immunization with the same clone as the target* W319C12 appears to be more sensitive to CMI than W319C6 (i.e. the opposite of what was found with NC cells); W324C17 is possibly slightly more sensitive than W324C57. For each clone there are no systematic differences in sensitivity between the cultured and mouse-passaged lines. There are also no systematic differences in cytotoxicity between spleen cells immunized with irradiated mouse-passaged cells ($I_1$), irradiated cultured cells ($I_2$) and viable cultured cells ($I_3$). Removing adherent cells from the effector population did not significantly affect the results with any of the targets tested.

*Cross immunization* In general, spleen cells from donors immunized with a different clone to the

**Table V** Long-term $^{51}Cr$ release with immunized effectors. W324 targets.

| Category of effector | E:T ratio | Value of ΔCI for target shown | | | |
|---|---|---|---|---|---|
| | | 324C17C | 324C17M | 324C57C | 324C57M |
| $C17I_1$ | 100 | 10.3 | 7.8 | 0.6 | 1.7 |
| | 50 | 19.0 | 6.1 | 0 | 3.2 |
| | 25 | 2.4 | 6.1 | | |
| $C17I'_1$ | 100 | 5.6 | 13.2 | 0 | 2.0 |
| | 50 | 7.6 | 5.3 | 0 | 3.6 |
| | 25 | | | | |
| $C17I_2$ | 100 | 4.8 | 3.1 | 3.9 | 1.7 |
| | 50 | 10.2 | 3.0 | 0 | 3.0 |
| | 25 | 0.4 | 0.7 | | |
| $C17I_3$ | 100 | 4.0 | 7.8 | 2.7 | 4.9 |
| | 50 | 10.1 | 0 | 3.8 | 8.4 |
| | 25 | 5.3 | 0 | | |
| $C57I_1$ | 100 | 1.4 | 4.0 | 0 | 1.6 |
| | 50 | 4.6 | 2.3 | 0.5 | 3.8 |
| | 25 | | | 0 | 0 |
| $C57I'_1$ | 100 | 0 | 0 | 1.3 | 6.2 |
| | 50 | 3.4 | 0 | 0.2 | 5.2 |
| | 25 | | | | |
| $C57I_2$ | 100 | 7.8 | 7.8 | 9.6 | 8.7 |
| | 50 | 14.6 | 5.6 | 2.0 | 6.9 |
| | 25 | | | 0 | 0 |
| $C57I_3$ | 100 | 5.2 | 5.0 | 0.6 | 2.0 |
| | 50 | 9.1 | 8.8 | 0 | 3.7 |
| | 25 | | | 0 | 0 |

See footnote to **Table IV** regarding the various categories of effectors and the definition of ΔCI.

target tested were either no more cytotoxic, or only slightly more cytotoxic, than normal spleen cells. An exception to this rule is the sensitivity in the IUDR assay, but not the chromium release assay, of 319C6M cells to spleen cells from mice immunized with C12M.

### Discussion

The primary object of the present experiments was to test the hypothesis that when cultured cloned murine fibrosarcoma lines are passaged in immunodeficient mice they acquire the capacity to grow in normal isogenic hosts because, during the initial passage, they beome resistant to attack by NK or NC cells, and this postulated resistance just sufficies to tip the balance in the favour when they encounter combined attack by NK/NC cells and cytotoxic T cells in the normal host.

It now seems clear that this hypothesis is false so far as NK cells are concerned because with all the clones tested even the cultured lines are NK cell resistant. Acquisition of resistance to NC cells, on the other hand, may play a role with some clones, e.g. W319C6. In the present experiments the cultured line of this clone was moderately NC cell sensitive and became less so on passage, and this accords with our previous finding (Woodruff & Hodson, 1985) that after s.c. injection of viable $^{125}I$-labelled cells to non-immunized mice, the rate of loss of label from day 1 to day 5 was greater with C6C than with C6MC. This will not serve as a general explanation however because 3 of the 4 cultured lines tested *in vitro* showed at most only slight sensitivity to NC cells. Moreover, we have shown recently with one other clone (W324C17) that insensitivity *in vitro* is associated with insensitivity *in vivo* as indicated by the slow early (day 1–day 5) loss of label after s.c. injection of $^{125}I$-labelled cells (Woodruff & Hodson, unpublished).

A subsidiary objective was to determine whether the conclusion drawn from our previous *in vivo* experiments (Woodruff & Hodson, 1985) that cultured and mouse-passaged lines of the same clone differ little, if at all, in their capacity to evoke an immune reaction, holds good also when the magnitude of the reaction is assessed by *in vitro* assays. It now seems clear that this is the case.

Of the three hypotheses proposed in our previous paper to account for the effect of passage in immunodeficient mice, two remain: the acquisition of a protective surface molecule, and the loss or modification of a Class I MHC molecule necessary for recognition by T cells. The fact that passaged tumour cells remain immunogenic tells against the latter possibility, but it is conceivable that the immunogenic stimulus might be provided by host T cells which have acquired TATA from the tumour and present these, in association with their own Class I MHC molecules, to other T cells.

Experiments designed to explore these and other possibilities will be reported in a subsequent paper.

We thank Mrs E. Clark for skilled technical assistance, Professor H.J. Evans for the privilege of working in this Unit, and the Medical Research Council, UK for a Project Grant.

### References

HERBERMAN, R.B., NUNN, M.F. & LAVRIN, D.H. (1975). Natural cytotoxic reactivity of mouse lymphoid cells against syngeneic and allogeneic tumours. I. Distribution of reactivity and specificity. *Int. J. Cancer*, **16**, 216.

KIESSLING, R. & WIGZELL, H. (1979). An analysis of the murine NK cell as to structure, function and biological relevance. *Immunol. Rev.*, **44**, 165.

STUTMAN, O., PAIGE, C.J. & FEO FIGARELLA, E. (1978). Natural cytotoxic cells against solid tumours in mice. I. Strain and age distribution and target cell susceptibility. *J. Immunol.*, **121**, 1819.

WOODRUFF, M.F.A. & HODSON, B.A. (1985). The effect of passage *in vitro* and *in vivo* on the properties of murine fibrosarcomas. I. Tumorigenicity and immunogenicity. *Br. J. Cancer*, **52**, 161.